# Fine mapping of a major QTL for awn length in barley using a multiparent mapping population

**DOI:** 10.1007/s00122-016-2807-y

**Published:** 2016-10-12

**Authors:** Corinna B. Liller, Agatha Walla, Martin P. Boer, Pete Hedley, Malcolm Macaulay, Sieglinde Effgen, Maria von Korff, G. Wilma van Esse, Maarten Koornneef

**Affiliations:** 1 Department Plant Breeding and Genetics, Max Planck Institute for Plant Breeding Research, Carl-von-Linné-Weg 10, 50829 Cologne, Germany; 2 Institute of Plant Genetics, Heinrich Heine University Duesseldorf, Universitätsstr. 1, 40225 Düsseldorf, Germany; 3 Cluster of Excellence in Plant Sciences (CEPLAS), Heinrich-Heine-Universität Düsseldorf, Universitätsstr. 1, 40255 Düsseldorf, Germany; 4 Biometris, Plant Research International, Wageningen University, Droevendaalsesteeg 1, 6708 PB Wageningen, The Netherlands; 5 The James Hutton Institute, Invergowrie, Dundee, DD2 5DA Scotland, UK; 6 Laboratory of Genetics, Wageningen University and Research, Droevendaalsesteeg 1, 6708 PB Wageningen, The Netherlands

## Abstract

**Key message:**

**Awn length was mapped using a multiparent population derived from cv. Morex and four wild accessions. One QTL was fine mapped and candidate genes were identified in NILs by RNA-seq.**

**Abstract:**

Barley awns are photosynthetically active and contribute to grain yield. Awn length is variable among both wild and cultivated barley genotypes and many mutants with alterations in awn length have been identified. Here, we used a multiparent mapping population derived from cv. Morex and four genetically diverse wild barley lines to detect quantitative trait loci (QTLs) for awn length. Twelve QTLs, distributed over the barley genome, were identified with the most significant one located on chromosome arm 7HL (QTL AL7.1). The effect of AL7.1 was confirmed using near isogenic lines (NILs) and fine-mapped in two independent heterogeneous inbred families to *a* < 0.9 cM interval. With exception of a small effect on grain width, no other traits such as plant height or flowering time were affected by AL7.1. Variant calling on transcripts obtained from RNA sequencing reads in NILs was used to narrow down the list of candidate genes located in the interval. This data may be used for further characterization and unravelling of the mechanisms underlying natural variation in awn length.

**Electronic supplementary material:**

The online version of this article (doi:10.1007/s00122-016-2807-y) contains supplementary material, which is available to authorized users.

## Introduction

The awn, developing as a long slender extension of the lemma in a grass spikelet, is a reduced leaf (Grundbacher 1963). In wild species, their function is thought to assist seed dispersal and protection (Elbaum et al. 2007). The awns of the cereal crops barley (*Hordeum vulgare*), wheat (*Triticum aestivum*), rye (*Secale cereale*) and oat (*Avena sativa*) have a similar anatomy: they have a triangular shape with the base directed towards the rachis; three vascular bundles; and two long strands of chlorenchyma, which contribute to the net photosynthetic activity of the spike (Grundbacher 1963; Li et al. 2006). A number of loci affecting awn length in barley are described and categorised in two main phenotypic groups: (1) loci that affect other traits such as plant height, leaf shape, and spike length; and (2) loci that mainly affect awn length or brittleness (Franckowiak and Lundqvist 2012; von Bothmer et al. 2003). Several genes from the first group have recently been shown to be involved in brassinosteroid (BR) biosynthesis or signalling (Dockter et al. 2014). The *short awn 2* (*lks2*) gene, which belongs to the second group [allelic to *breviaristatum* (*ari)* -*d* and *unbranched style 4* (*ubs4*)] is located on the long arm of chromosome 7H and was found to encode for a SHORT INTERNODES (SHI)-type transcription factor (Yuo et al. 2012).

In general, awned barley genotypes outyield awnless isogenic lines, especially under warm and dry conditions (Bort et al. 1994; Grundbacher 1963; Scharen et al. 1983, Rebetzke et al. 2016). Within the spike the awns are the main contributors to the photosynthetic activity (Abebe et al. 2009). Consequently, the absence of awns reduces seed weight and size because of a reduction in starch content (Bort et al. 1994; Grundbacher 1963; Jiang et al. 2006; Li et al. 2006; Scharen et al. 1983). Given the potential impact of awn length on yield in barley cultivars, it is important to identify novel QTLs and genes that affect this trait. Previous genetic studies described QTLs for awn length on chromosome 3H (Chen et al. 2012; Gyenis et al. 2007; Wang et al. 2010), possibly co-localizing with either *ari*-*a* or *semi*-*brachytic* (*uzu*) (HvBRI1); on 6HS (Sun et al. 2011); close to the row type locus *six*-*rowed spike 1* (*Vrs1*) on 2H (Sameri et al. 2006), where the awnless locus *Lks1* has been mapped; on 7H (Sameri et al. 2006), possibly co-localizing with either *ari*-*f* or *ari*-*n* (Druka et al. 2011; Franckowiak and Lundqvist 2012); and on 7HL which is associated with DIMINUTO (*HvDIM*) (Dockter et al. 2014). The majority of these studies have investigated the genetic basis of awn length differences in cultivated barley. Substantial variation in awn length, however, is also observed in wild barley (Badr et al. 2000) which is characterised by higher genetic diversity than cultivated barley and thus represents a valuable source to improve the elite barley genepool (Russell et al. 2004). Therefore, we generated a multiparent intercross population between wild and cultivated barley to map and identify the genetic basis for differences in awn length. The advantages of using multiparent populations compared to classical bi-parental populations are that: (1) several alleles and their interaction with different backgrounds can be analysed simultaneously and (2) QTLs can be detected with higher precision and resolution due to the increased level of recombination (Cavanagh et al. 2008). We generated a multiparent mapping population by intercrossing cv. Morex, three ssp. *spontaneum* from the Fertile Crescent and one ssp. *agriocrithon* from the Tibetan highlands in China. After one round of backcrossing to the recipient Morex to reduce the effect of deleterious wild alleles, the resulting BC1F1 was intercrossed in all possible combinations to generate by single seed descent 6 subpopulations with 130–150 RILs each. The QTL analysis resulted in the identification of a major QTL for awn length on 7HL where the wild barley allele decreased awn length. Fine mapping of this locus using independent heterozygous inbred families (HIFs) led to a delimitation of the QTL locus to <0.9 cM. The position of the locus was verified using mapping of the variants between RNA sequencing reads obtained from two near isogenic lines differing only in the QTL region. The major locus on 7HL was not associated with any other traits nor did it map to previously identified awn length genes located in this region such as *HvLks2* or *HvDIM*.

## Materials and methods

### Plant material

The multiparent recombinant inbred Line (RIL) mapping population was constructed through crosses between the barley cultivar Morex and four wild barley lines selected for large genetic variation (Badr et al. 2000). Three wild barley genotypes originated from the eastern and western Fertile Crescent, the centre of barley domestication [ssp. *spontaneum*: HID 4 (A), Iraq; HID 64 (B), Turkey; and HID 369 (C), Israel and one barley genotype originated from the Tibetan highlands in China (ssp*. agriocrithon*: HID 382(D)]. Each wild barley line was crossed to cv. Morex (ssp. vulgare, USA) and the F1 hybrids were backcrossed to Morex once. These backcross generations were intercrossed with each other at every possible combination, resulting in six independent subpopulations. From these intercrosses, 916 recombinant inbred lines (RILs) with 130–165 lines per subpopulation were made by single seed descent (SSD) (Online Resource 1) for six generations. The resulting F6 generation was used for genotyping and QTL mapping.

### Plant growth conditions

For QTL mapping of awn length, the F6 generation was grown with four replicate plants per RIL under outdoor conditions. Plants of the multiparent RIL population were sown mid-January 2011 in 96-well trays, germinated under greenhouse conditions and placed outdoors after the first leaf emerged (end of January). Plants were transferred (mid-Feb) to 10 L pots, each pot containing one plant. Pots were arranged in 15 rows spaced 1 m apart, each row contained 61 pots with a distance of 15 cm. The entire plot was divided into two blocks, two replicate plants per genotype were randomised together within each block. The plot was surrounded by one row of border pots containing elite barley cultivars. The plants were irrigated using a sprinkler robot. For QTL confirmation and additional experiments performed in 2012, 2013 and 2014 the plot was set-up using 12 L pots as described previously (Liller et al. 2015) only in this study the plot was divided into two blocks.

A custom-made peat and clay soil mixture (EinheitsErde^®^ ED73 Osmocote, Einheitserdewerke Werkverband e.V., Sinntal-Altengronau, Germany) containing a long term fertilizer was used for the outdoor and greenhouse grown plants. Greenhouse experiments were performed in an air-conditioned greenhouse under long day conditions (16 h, 22 °C day; 8 h, 18 °C night) using either 0.3 or 0.5 L pots. In all experiments, additional fertilizer and/or pesticides were applied to the plants when necessary.

### Awn length measurement

All spikes used for awn length measurements were collected at maturity. The spikes were dried in a drying cabinet (Heraeus) at 37 °C for at least 3 days. Awn length (length of the detached longest awn of the spike) was measured using a 200 mm digital calliper (Mitutoyo Deutschland, Neuss) The population involves crosses between 2-rowed HID lines and 6-rowed Morex, and thus also segregates for row-type. In the six-rowed plants, the awn length may differ between the central spikelet and the lateral spikelet. Therefore, only the awn length of the central spikelet was used for QTL mapping. At least four independent biological replicates (*n* ≥ 2 technical replicates) were used to obtain an average awn length used for QTL analysis.

### DNA extraction

DNA was extracted from fresh or freeze-dried leaf material using the Qiagen BioSprint following the manufacturer’s protocol (Qiagen, Hilden, Germany). DNA was eluted in 150 µL supplied buffer AE and concentration was measured using the NanoDrop spectrophotometer (PEQLAB, Erlangen, Germany).

### QTL mapping

One individual of each RIL was genotyped with a selection of 384 BOPA single-nucleotide polymorphism (SNP) markers from the barley Consensus Map (Close et al. 2009) at the James Hutton Institute (Dundee, Scotland, UK) using the Illumina Golden Gate Assay system.

The markers were assigned to their positions on the POPSEQ map (Ariyadasa et al. 2014). The SNP markers were used to calculate the genetic predictors, i.e. the probabilities that a particular RIL at a given locus has the genotype of one of the five founders. The founder to offspring probabilities were calculated using a Hidden Markov Model (Rabiner 1989; Zheng et al. 2015), an extension of the inheritance vector approach as described in Huang et al. (2011).

The QTL-mapping was done using linear mixed models in Genstat 17 (VSN international). In the first step of the QTL analysis we used a genome scan, known as simple interval mapping (SIM) (Lander and Botstein 1989). Let $\underline{y}_{ik}$ be the awn length for RIL *i* in cross *k*, then the following model can be given:

1 $$\underline{y}_{ik} = \mu_{k} + x_{ikl}^{T} \underline{\beta }_{l} + \underline{\varepsilon }_{ik}$$

where $\mu_{k}$ is a fixed effect, the vector $x_{ikl}^{{}} = (x_{ikl1} ,x_{ikl2} , \ldots ,x_{ijl5} )^{T}$ contains the probabilities that RIL *i* in cross *k* for locus *l* has the genotype of wild barley line *f* (*f* = 1,…,4). The vector $\underline{\beta }_{l}$ is a 4-dimensional vector of random founder effects corresponding to locus *l*. Finally, $\underline{\varepsilon }_{ik}$ is the residual error for RIL *i* in cross *k*, with cross-specific variance $V\left( {\underline{\varepsilon }_{ik} } \right) = \sigma_{\varepsilon ,k}^{2}$.

In the second step, we ran a genome scan using a multi-QTL model adjusting for background QTLs. The background QTL with the strongest effect was first added, then a new QTL-scan was performed to detect the second strongest QTL, and this procedure was repeated until no new QTLs were detected above the QTL significance threshold of −log10(*P*) = 3.2 (Huang et al. 2011). Each background QTL was modelled as a random effect, and the model is an extension of Eq. (1):

2 $$\underline{y}_{ik} = \mu_{k} + \sum\limits_{{c \in {\kern 1pt} C}} {x_{ikc}^{T} \underline{\beta }_{c} } \; + \;x_{ikl}^{T} \underline{\beta }_{l} \; + \;\underline{\varepsilon }_{ik}$$

where C is a set of co-factors to correct for QTLs elsewhere in the genome, and *l* is the putative QTL. Co-factors within 10 cM of the putative QTL were excluded.

In the final step of the QTL-analyses all QTLs detected with model (2) were included in the model:

3 $$\underline{y}_{ik} = \mu_{k} + \sum\limits_{q \in Q} {x_{ikq}^{T} \underline{\beta }_{q} } + \underline{\varepsilon }_{ik}$$

where Q is the set of selected QTLs. For each QTL the min log10(*P*)-values, the founder effects and the standard errors were estimated. Epistatic interactions between the 12 QTL were tested pairwise according to Huang et al. (2011).

### QTL confirmation and fine mapping

For QTL confirmation, offspring of RILs heterozygous at the most significant QTL (QTL AL7.1) in the *F*_6_ generation was evaluated. These RILs represent Heterogeneous Inbred Families (HIFs) as defined by Tuinstra et al. (1997) and were genotyped with markers closest to the putative QTL. For the major awn length QTL on chromosome arm 7HL (AL7.1) 23–26 HIFs were grown under outdoor conditions in 2012. Genotyping was carried out with the markers 3_1489 and 2_0117 which are located close to the putative awn length QTL. Subpopulations were abbreviated based on the parents segregating at AL7.1, defining Morex (M) and alleles of the wild barleys HID 4 (A), HID 64 (B), HID 369 (C) and HID 382 (D) resulting in the possible allele combinations AM, BM, CM and DM.

The selfed progeny of, respectively, 475 heterozygous plants of the HIFs BM1 and 452 of DM1 were used for fine mapping. Recombinants were selected using the flanking markers 3_0593/1_0999 for BM1 and 2_0483/1_0999 for DM1 resulting in 47 and 13 recombinant lines, respectively. All recombinant and seven non-recombinant plants per genotype were transplanted to large pots, genotyped with additional newly developed CAPS markers (Figs. 4, 5) located within the region of AL7.1 and grown to maturity. The HIFs were fixed for the other QTLs, thus the BM1 and DM1 HIFs used for fine mapping only segregated at AL7.1. For confirmation and further delimitation of the QTL interval, homozygous lines were selected from the (heterozygous) recombinant plants of populations BM1 and DM1 and phenotyped for awn length under outdoor conditions (Online Resource 2). Linkage maps for chromosome 7HL in the BM1 and DM1 populations were constructed using JoinMap 4 (Kyazma B.V., Wageningen, The Netherlands, https://www.kyazma.nl/index.php/JoinMap/). Polymorphic SNP markers were designed as CAPS/dCAPS markers (Konieczny and Ausubel 1993; Neff et al. 1998) based on SNPs described by Close et al. (2009), the Barley Consensus Map and the iSelect chip (http://bioinf.hutton.ac.uk/iselect/app/). CAPS/dCAPS sites were identified using dCAPS finder (http://helix.wustl.edu/dcaps/dcaps.html) (Neff et al. 1998). A list with primer sequences used for fine mapping can be found in Online Resource 3. PCR was performed in a total reaction volume of 10 µL containing 20–40 ng genomic DNA, 1× supplemented buffer, 0.5 µM forward and reverse primer, 15 µM dNTPs, 0.25 U *T*aq polymerase (Ampliqon, Odense, Denmark) and 5% glycerol or 2 mg BSA if necessary. Amplification was done using a touchdown (TD) PCR with an initial 3 min denaturation at 95 C, 15 TD cycles (30 s at 95 °C, 30 s TD (−1 °C per cycle) from 67 °C to 52 °C and 30 s at 72 °C) and 20 cycles with the 52° C annealing temperature. PCR products were digested with the respective restriction enzyme (New England Biolabs, Ipswich, MA, USA) according to manufacturer’s instructions. Bands were visualised on a 2.5 or 4% agarose gel containing 4 × 10^−5^ vol/vol % ethidium bromide.

### RNA extraction and sequencing

Homozygous near isogenic lines (NILs) derived from the (recombinant) DM1 HIF line -e35R harboring either the HID 382 (D) or Morex (M) allele at the QTL position were grown in the greenhouse under long day conditions (16 h, 22 °C day; 8 h, 18 °C night). Shoot apices were staged according to the Waddington scale (Waddington et al. 1983) and apex tissue was collected at stage 5.5 by pooling ten apices from homozygous D or M plants and immediate freezing in liquid nitrogen. RNA was extracted using the Qiagen RNeasy Plant Mini Kit (Qiagen, Hilden, Germany) following the manufacturers protocol and stored at −80 °C after DNase treatment (Ambion, Carlsbad, USA). The quality of the RNA was evaluated before library preparation using a bioanalyzer (Agilent). The Illumina TruSeq libraries were prepared using the manufacture’s protocol (version 2, Illumina). Single end sequencing was performed on the HiSeq 2000 (Illumina^®^) platform. For each library a minimum of 15 million reads were generated by multiplexing eight libraries. The initial quality control of the raw reads was performed using the FastQC software. Reads were trimmed using the Trimmomatic platform, embedded within the trinity pipeline (Grabherr et al. 2011; https://github.com/trinityrnaseq/trinityrnaseq/wiki), using the following default criteria: phred 33, leading and trailing 3, sliding window 4:15 and a minimum read length of 36. Sequence alignments were performed by Bowtie2 (version 2.1.0; http://bowtie-bio.sourceforge.net/bowtie2/index.shtml) using a merged dataset of High Confidence (HC) and Low Confidence (LC) predicted barley genes (The International Barley Genome Sequencing Consortium 2012) as reference. SAMtools (Li et al. 2009, version 1.2; http://www.htslib.org/) was used for BAM format conversion, sorting and indexing and read duplicate removal was conducted using the Picard command-line tool MarkDuplicate (version 1.110; https://broadinstitute.github.io/picard/command-line-overview.html). To correct misalignments, the Genome Analysis ToolKit (GATK, version 3.1) realigner was used with recommended settings. Variants were obtained using the GATK UnifiedGenotyper platform (minimum phred score of 30). Refinement of the variants was performed with the GATK Variant Filtration tool (Fisher Strand values FS >30.0; Qual By Depth values QD <2.0) to reduce false positive SNPs. Resulting SNP calls were kept for further analysis if they passed the filtration step and their read coverage exceeded four reads. Transcripts containing filtered homozygous SNPs were mapped to their respective position along the POPSEQ map of barley with R (The International Barley Genome Sequencing Consortium 2012). For expression analysis, the reads were aligned to the high confidence (HC) and low confidence (LC) gene set as described above and only in this case, the read duplicates were not removed from the BAM file. Raw counts were extracted from the BAM file using Salmon (Patro et al. 2015). Differentially regulated reads were called with a false discovery rate of 0.05 using the R bioconductor package limma-vroom (Ritchie et al. 2015). All RNA sequencing data has been submitted to the GEO database (GSE85338). For phylogenetic studies, the amino acid sequence of candidate MLOC_54434.1 was used as a query string to perform a sequence blast against the NCBI nr database of following organisms: *Hordeum vulgare* (taxid:4513), *Oryza sativa* (taxid:4530), *Brachypodium distachyon* (taxid:15368), *Sorghum bicolor* (taxid:4558*), Arabidopsis thaliana* (taxid:3702). Putative orthologues proteins were identified with a cutoff of *e* value 5*e*−10. The multiple alignment of chosen sequences was performed with CLUSTAL O (1.2.2) and a phylogenetic tree was inferred using the Neighbor-Joining method in MEGA5.

### Effect of awn length on grain filling

Plants of cv. Morex were grown under outdoor conditions. Upon heading, the plants were randomly assigned to three groups of equal size (*n* = 7) and all emerging spikes of these plants were treated as follows (latest 3 days after heading): (1) only the very tips of the awns were removed with scissors (full awn), (2) the awns were cut in half (half awn), or (3) the awns were cut off completely (no awn). This treatment was repeated with every emerging spike until the last spike had emerged. The plants were then allowed to reach full maturity without further disturbance and harvested individually. The cleaned seed (three technical replicates of 5 mL per biological replicate) was measured with the MARVIN Seed Analyser (GTA Sensorik, Neubrandenburg, Germany) to obtain seed width, length and area and thousand grain weight (TGW).

To assess the effect of the differences in awn length observed in the HIFs, seed from homozygous individuals from two mapping families (BM1 and DM1) obtained in the 2012 and 2014 outdoor experiments was cleaned and seed traits were also measured with the MARVIN Seed Analyser.

## Results

### QTL mapping of awn length in the multiparent RIL population

**Fig. 1:**
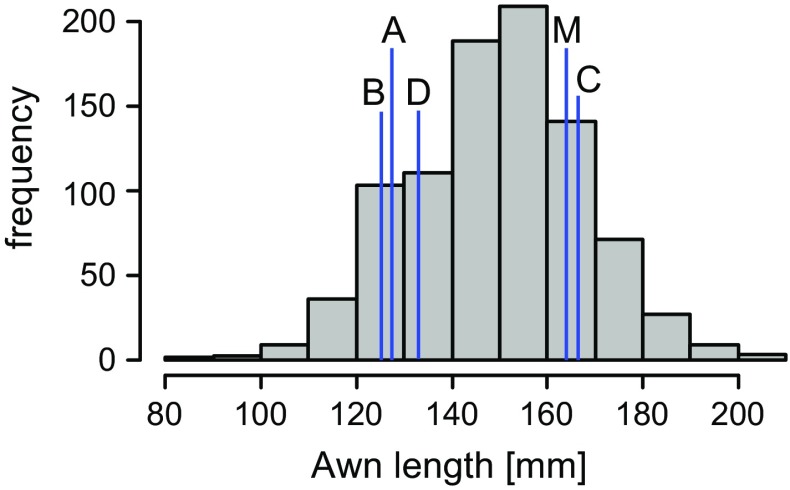
Distributions of awn length in the mapping population. Histograms of average awn length of RILs from the mapping population. The entire mapping population consists of 916 RILs. *Bars* mark averages of parental *lines* (*A* HID 4, *B* HID 64, *C* HID 369, *D* HID 382, *M* morex)

**Fig. 2:**
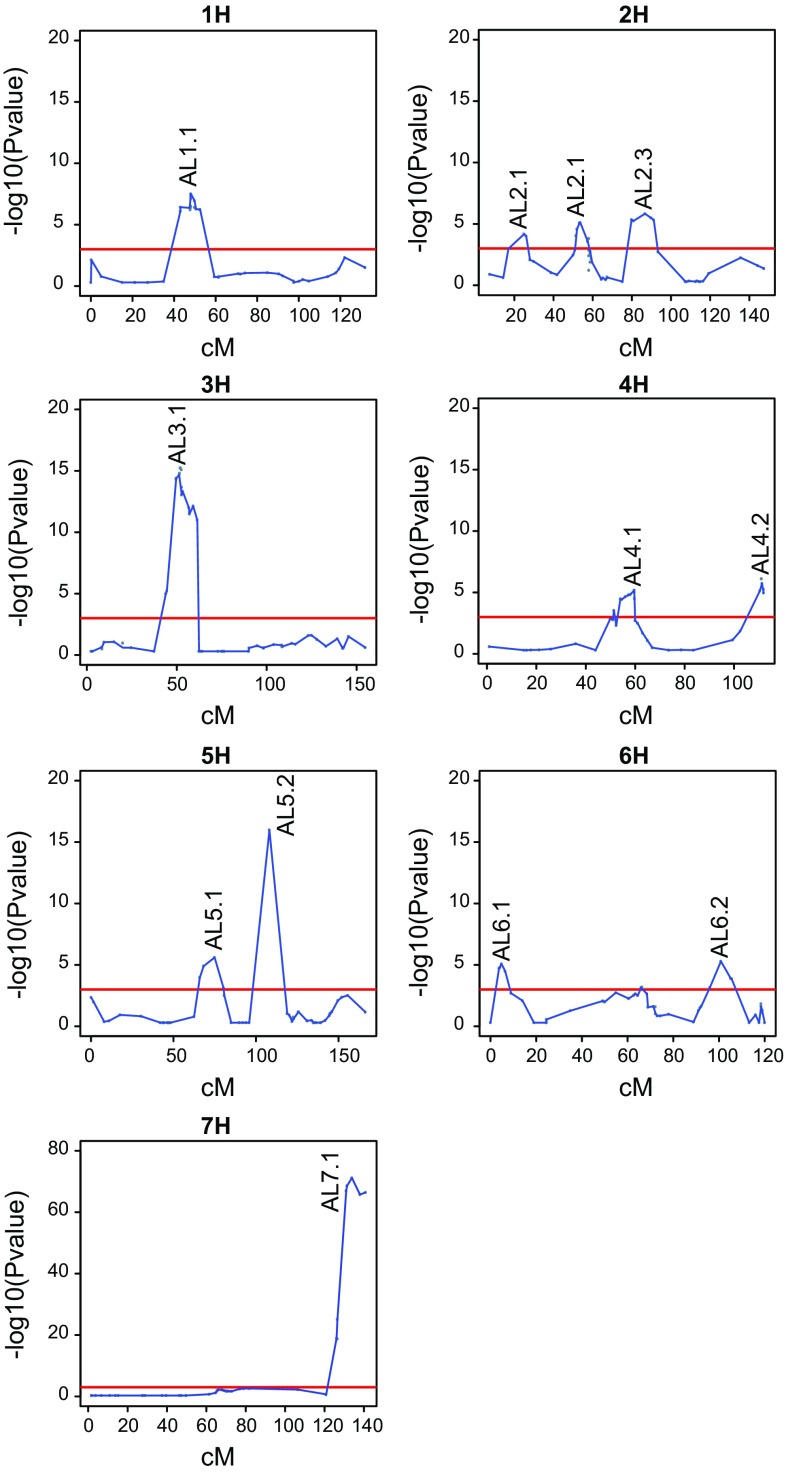
QTL mapping for awn length. Results of multiple-QTL mapping (MQM) of awn length on the multiparent RIL population. *Lines* show *p* values over the seven barley chromosomes. The *horizontal line* indicates the confidence threshold (−log10(*p*) = 3.2)

The multiparent RIL population used in this study was constructed from three lines of ssp. *spontaneum* originating from different parts around the Fertile Crescent (Iraq, Turkey and Israel) and one line of ssp. *agriocrithon* from the Tibetan highlands (China) crossed with the cv. Morex. To evaluate if the parents of this population differ in awn length we measured awn length of plants grown under outdoor conditions. The parents of the multiparent mapping population differed from one another in awn length, with HID 4 (Iraq), HID 64 (Turkey) and HID 382 (China) having significantly shorter awns when compared to cv. Morex (USA) and HID 369 (Israel) (Table 1). In the whole mapping population the trait showed a normal distribution ranging from 73.4 to 230.0 mm per RIL, including transgressive segregation in both directions and had a broad sense heritability of *H* ^*2*^ = 0.75 (Figs. 1, 2; Table 1, Online Resource 4). Genotyping with 384 BOPA single-nucleotide polymorphism (SNP) markers resulted in a total of 365 polymorphic markers in the entire population, with about 200–230 polymorphic markers in each of the subpopulations (Online Resource 5). A total of 12 significant QTLs (LOD >3) for awn length were identified using the whole mapping population (Table 2, Online Resource 6). The major QTL for awn length is located at position 133.9 cM on chromosome 7H (−log10(*p*) = 71.2). The allele effects of the four wild barley parents when compared to Morex were determined for each QTL. From each parent, QTL with positive as well as negative effects were identified (Table 2). No interactions between any of the 12 QTL were significant after multiple testing correction and we conclude that the loci act in an additive way.

**Table 1:** Awn length of parental lines and all RILs of the multiparent RIL population

	Parental lines					All RILs		*H* ^2^
	Morex	HID 4	HID 64	HID 369	HID 382	Average	Range	
Awn length (mm)^c^	164 ± 16.8^a^	127 ± 7.6^b^	125 ± 10^b^	166 ± 6.2^a^	132 ± 7.6^b^	150 ± 22.3	73–230.0	0.75

Letters a and b indicate significant differences (*p* ≤ 0.05) between the parental lines, determined with a One-way ANOVA using a Tuckey HSD as post hoc test

^c^Average awn length values mm ± STDEV, for parental lines *n* ≥ 6 awns

**Table 2:** Positions of QTLs for awn length on the barley genetic map and their allelic effects (in mm)

QTL	chr^a^	pos^b^	Marker			−log10 (*p*)	Allele effect ± SE^c^			
			Peak	Flanking 1	Flanking 2		HID 4	HID 64	HID 369	HID 382
AL1.1	1H	48.1	2_0798	2_0617	2_0956	7.5	4.0 ± 1.6	6.7 ± 1.2	1.9 ± 1.3	3.9 ± 1.5
AL2.1	2H	25.0	2_1261	1_0180	2_0864	4.2	−3.8 ± 1.6	−3.1 ± 1.4	−4.3 ± 1.6	−4.9 ± 1.6
AL2.2	2H	53.7	1_0325	1_1505	2_0585	5.1	7.5 ± 1.8	−1.1 ± 1.6	−4.4 ± 1.6	2.4 ± 1.6
AL2.3	2H	86.8	1_0214	1_0786	1_1480	5.8	5.0 ± 1.7	0.8 ± 1.7	0.0 ± 5.2	6.8 ± 1.4
AL3.1	3H	52.0	1_0456	1_0081	1_0172	15.2	−6.4 ± 1.7	−5.9 ± 1.5	−10.6 ± 1.5	1.8 ± 1.5
AL4.1	4H	59.6	2_0924	1_1042	1_1224	5.2	2.6 ± 1.6	10.4 ± 2.9	5.1 ± 1.2	0.1 ± 1.6
AL4.2	4H	111.1	1_1066	1_0334	2_0553	6.1	−2.3 ± 1.5	5.1 ± 2.1	−4.0 ± 1.5	4.8 ± 1.2
AL5.1	5H	74.9	2_1150	1_0167	2_0526	5.6	−4.4 ± 2.7	−2.6 ± 2.1	−3.2 ± 1.6	−6.5 ± 1.4
AL5.2	5H	108.1	1_0094	2_0805	2_1203	16.0	9.6 ± 1.8	4.5 ± 1.7	18.5 ± 2.6	8.7 ± 2.1
AL6.1	6H	4.8	2_0882	2_0292	2_0262	5.1	−3.3 ± 1.8	−8.4 ± 1.7	0.0 ± 5.5	1.1 ± 1.6
AL6.2	6H	100.8	2_0036	1_0015	1_0175	5.3	5.6 ± 1.7	5.2 ± 1.3	3.3 ± 3.3	−0.5 ± 1.4
AL7.1	7H	133.9	2_0962	2_1104	End	71.2	−3.5 ± 2.0	−19.0 ± 2.0	−1.0 ± 2.1	−24.7 ± 1.3

^a^Chromosome number

^b^QTL position (cM) of the peak marker

^c^Additive effects (in mm) of the wild barley alleles compared to Morex (±standard error). The positive or negative value indicates that the wild barley allele increases or reduces the trait, respectively

### Fine mapping of the major QTL on 7H

**Fig. 3:**
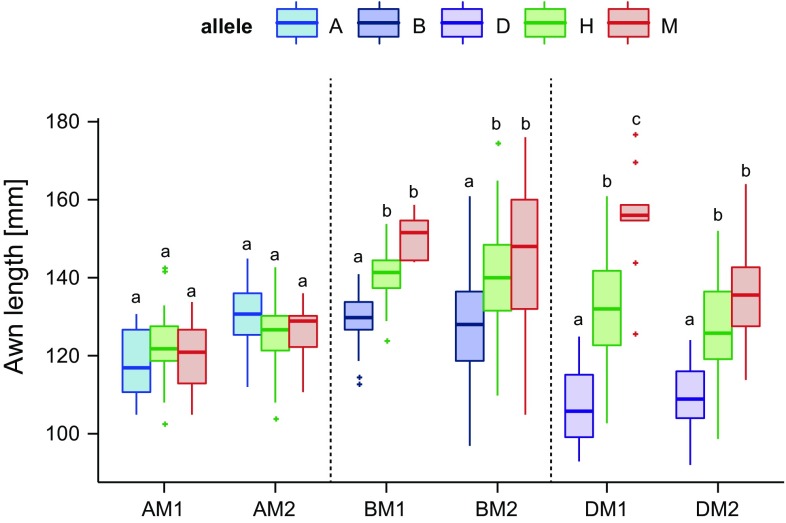
Boxplots of awn length of six HIFs segregating for the QTL on chromosome arm 7HL. For each allele combination (AM, BM, DM) two HIFs were selected that segregate for the cultivated barley (Morex) and alleles of the wild barleys. *A* HID 4, *B* HID 64, *D* HID 382, *M* Morex, *H* heterozygous. Different *small letters* indicate significant differences between genotypes (one-way ANOVA, *p* ≤ 0.05, *n* ≥ 10)

AL7.1 on chromosome arm 7HL was the most significant QTL, therefore, it was selected for fine mapping. The major advantage of heterozygous inbred families (HIFs) is that it enables fine mapping of the QTL while comparing the trait in an otherwise isogenic background (Tuinstra et al. 1997). Two HIFs segregating for cultivated barley (Morex, M) and alleles of the wild barleys HID 4 (A), HID 64 (B) and HID 382 (D) were selected for each allele combination (AM, BM, DM). HID 369 (C) was not included in fine mapping, as it was not significantly different in awn length from Morex (Table 1). The HIFs were fixed for the wild allele of *vrs1* resulting in a 2-rowed spike. The segregating families were grown under outdoor conditions and genotyped with marker 3_1489 located close to the peak of AL7.1. The two families segregating for the AM allele combination did not exhibit significant differences in awn length between the genotypes, which is consistent with the relatively small allele effect of parent HID 4 (Table 2). In all tested families segregating for the B and M, or D and M alleles at 3_1489, the homozygous HIFs differed significantly (*p* ≤ 0.05) in awn length from one another (Fig. 3). In each case, the Morex allele increased awn length compared to the wild barley allele, which was consistent with the allele effect estimated for this QTL (Table 2). In the BM and DM populations the heterozygotes were intermediate to the homozygote genotypes indicating that the two alleles have incomplete dominance (Fig. 3).

**Fig. 4:**
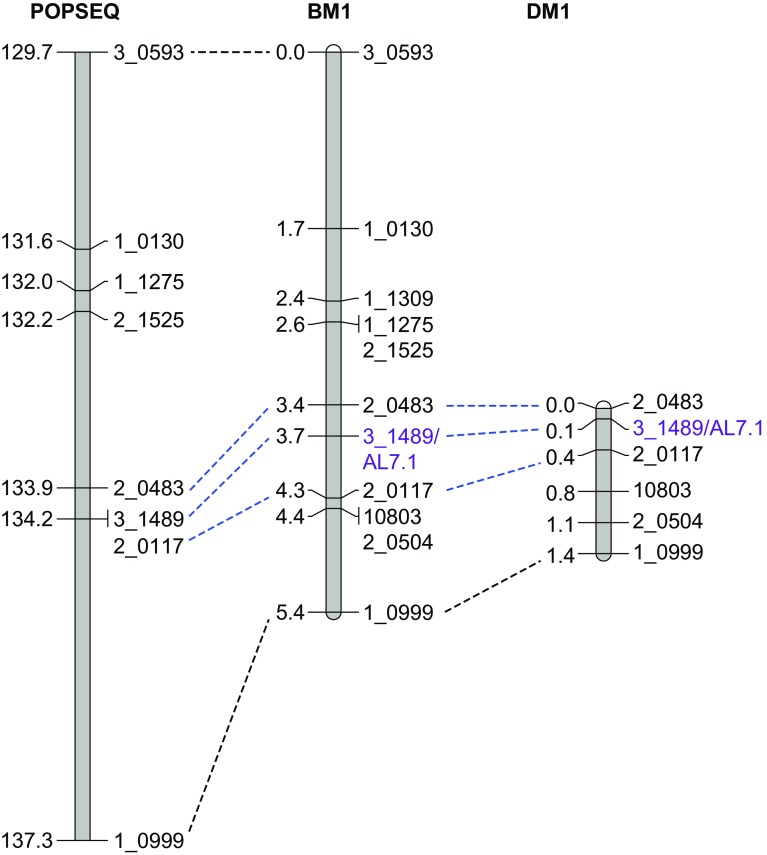
Fine mapping of BM1 and DM1. Mini map derived from the BM1 and DM1 segregating families, and position of the markers on the POPSEQ Map (Ariyadasa et al. 2014) that were used to construct linkage maps of the QTL region in BM1 and DM1. Marker 3_1489 (purple) completely co-segregates with AL7.1. From family BM1, a map between markers 3_0593 and 1_0999 could be reconstructed corresponding to 5.4 cM. Family DM1 segregated only between markers 2_0483 and 1_0999 with a genetic distance of 1.4 cM

One family from each of the confirmed allele combinations was selected for fine mapping (BM1 and DM1). Both families segregated for the wild barley and Morex alleles according to Mendelian ratios and exhibited recombination events between the flanking markers used (Online Resource 7). Recombinant plants were genotyped with additional markers to determine the recombination breakpoints (Online Resource 8) and the genotype data were used to construct linkage maps of the QTL region for both families (Fig. 4).

**Fig. 5:**
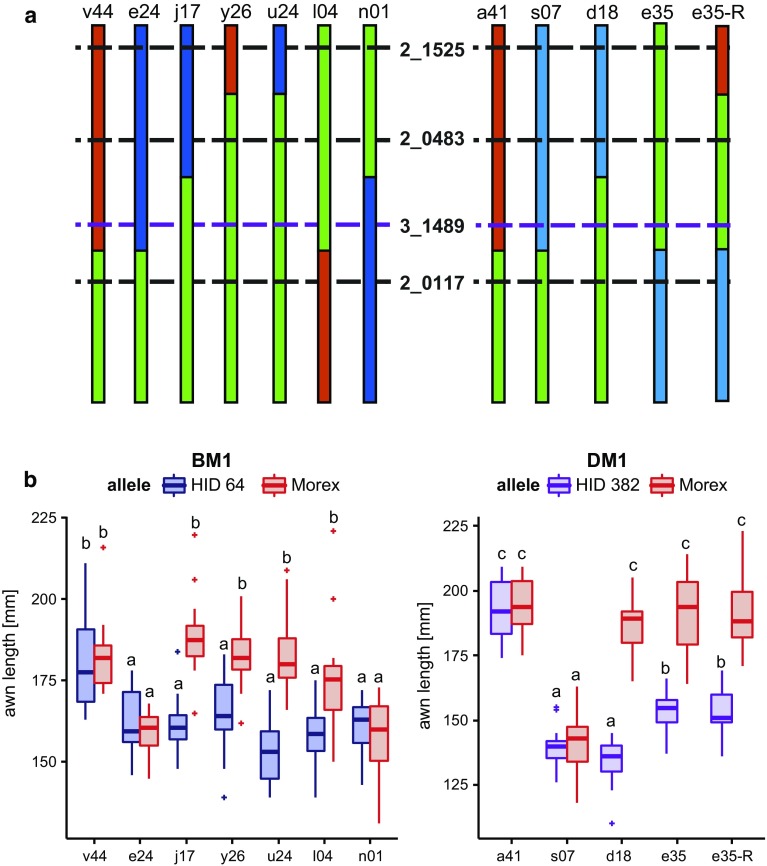
Selection of HIFs used for QTL confirmation. **a** Graphic representation of recombinants used for QTL fine-mapping. *Black horizontal bars* represent positions of tested markers. *Purple dashed line* position of the most significant QTL marker; *black dashed lines* positions of markers just outside the QTL region. Colour code *red* Morex, *blue* wild barley, and *green* heterozygous. (B) Boxplots of awn lengths collected from recombinant homozygous NILs (*B* HID 64, *D* HID 382, *M* morex.). Different *small letters* indicate significant differences between the genotypes (one-way ANOVA, *p* ≤ 0.05, *n* ≥ 6)

All recombinant plants and selected non-recombinant plants were grown to maturity and phenotyped for awn length. The non-recombinant plants exhibited similar differences in awn length as observed in the previous generation (Online Resource 9). The NIL progenies of all recombinants (Online Resource 8) were genotyped for markers between the two markers flanking the QTL region and awn length was measured for all plants allowing a comparison of the effect of Morex, HID 64 (B) and HID 382 (D) for each marker. Similar to earlier mapping experiments awn length inherited in an incomplete dominant manner with the Morex allele increasing awn length compared to the wild barley alleles (Online Resource 10,11 and 12). Fine mapping showed that AL7.1 is located between markers 2_0483 and 2_0117 for population BM1 as well as for DM1 (Fig. 5, Online Resource 8). This reduced the QTL interval to less than 1 cM (observed map distance: 0.9 cm in BM1 and 0.4 cM in DM1, respectively) and did not recombine with marker 3_1489 in all tested individuals.

Awn length allelic variants are often associated with pleiotropic effects on other traits such as tillering and or plant height. Therefore, tiller number, spike length, plant height and flowering time were measured in the HIFs exhibiting the clearest differences in awn length (BM1, DM1 and DM2). The homozygous HIFs did not exhibit a significant difference in any of these traits (Online Resource 13). To evaluate the novelty of AL7.1 we performed an in silico mapping using markers that were previously associated with awn length genes (Online Resource 14). Two awn length related QTLs are located on 7HL, the SHORT INTERNODES (SHI)-family transcription factor encoded by the *HvLks2* gene, and the brass GATK VariantFiltration tool gene. AL7.1 is located 30 cM distal of *HvLks2*, close to *HvDIM* which maps to 140.3 cM on the POPSEQ map. Neither *HvLks2* nor *HvDIM* are located in between the markers flanking AL7.1. Taken together, we have fine mapped an awn length QTL in the distal region of chromosome arm 7HL that does not map to any previously described awn length locus. In addition, AL7.1 did not exhibit any pleiotropic effect on the other measured traits.

### RNA sequencing of NILs corroborates fine mapping

**Fig. 6:**
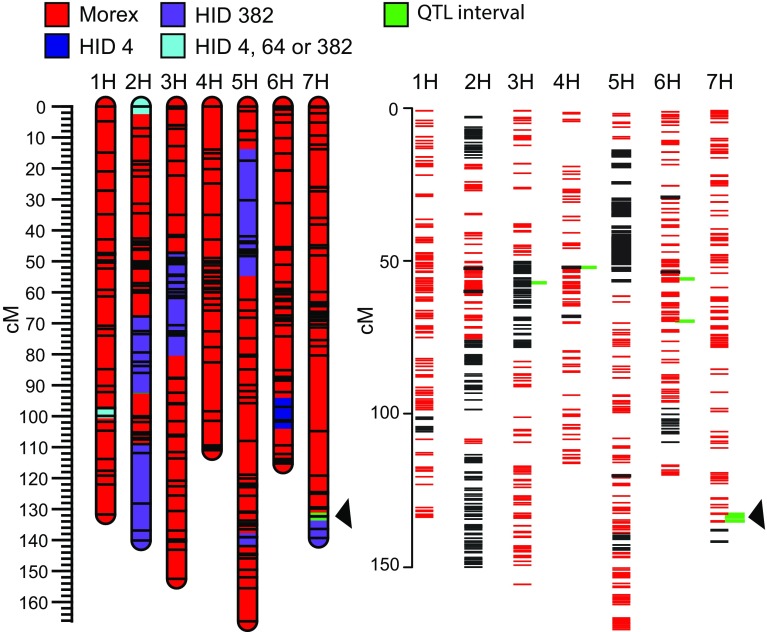
Comparison between the DM1-e35R NILs. The major QTL on 7HL associating with awn length is marked with an *arrow head*. *Left panel* shows the introgressions of the wild barley into the cv. Morex. *Right panel* shows expressed variants between wild HID *lines* (*black*) and Morex (*red*). *Green bars* indicate the variants polymorphic between the two DM1-e35R NILs, homozygous for, respectively, the Morex and HID382 (D) allele

To verify the fine mapping of AL7.1 we performed RNA sequencing, using next generation sequencing on the homozygous NIL pair derived from the heterozygous recombinant DM1-e35R HIF line. RNA was obtained from shoot apical meristem tissue at Waddington stage (W) 5.5 (Waddington et al. 1983), at this time point the stylar canal starts to close and the first awns are emerging. Transcripts exhibiting a SNP when compared to the reference sequence (cv. Morex) were mapped to the barley POPSEQ map. A total of 1295 genes can be assigned to derive from the introgressed wild barleys, while 3445 genes show the Morex allele. The number of Morex alleles accounts for 72%, reflecting the expected value of 75% of the Morex genome in the HIF progenies. Direct comparison of the SNPs between the DM1- e35R NILs confirmed the position of the segregating region on chromosome arm 7HL, as well as the introgression pattern derived from the initial genotyping data (Fig. 6).

RNA sequencing can also be used to narrow down the number of candidate genes, which comprise differentially regulated genes, as well as transcripts with nonsynonymous SNPs. The segregating region on chromosome 7HL identified with RNA sequencing spans an interval of 2.2 cM (132.01–134.21 cM) according to the POPSEQ map (Online resource 15). This interval contains 123 genes (43 HC and 80 LC genes). The 0.9 cM interval identified through fine mapping is completely located within this 2.2 cM interval and contains 66 genes (26 HC, 40 LC). Within this interval, transcripts were detected for 19 out of the 66 genes and only eight transcripts exhibited polymorphisms between the DM1-e35R NILs (Online resource 15). Analysis of transcripts with no assigned position according to the POPSEQ map revealed three additional genes containing polymorphisms. Expression analysis performed on the DM1-e35R NILs indicated that two genes (MLOC_72272.1 and MLOC_54434.1), both located within the introgression, were differentially regulated between the DM1-e35R NILs (adjusted *p* value ≤0.05) (Online resource 15). The low number of differentially regulated genes identified between the two NILs may be caused by the high variation observed between the individual biological replicates (Online resource 16).

Because MLOC_54434.1, encoding a zinc finger protein 3, also revealed a polymorphism in the variant calling, we performed Sanger sequencing of MLOC_54434.1 in the BM1 and DM1 NILs. The NILs that exhibited a reduced awn length contained an AG insertion at position 37. This insertion causes a frameshift in the protein sequence and introduces a premature stop codon. The marker developed based on the detected variant in MLOC_54434.1, which is located at 134.21 cM in the POPSEQ map, co-segregated with the phenotype of the recombinant homozygous NILs from the two mapping populations BM1 and DM1 (Online resource 15). Assuming that the causative gene is expressed in the selected developmental stage, we consider this zinc-finger TF as prime candidate gene for the awn length phenotype. Phylogenetic analysis revealed no homology with known zinc-finger domain containing awn length genes *HvLks2* and *OsDL* (Online resource 17). Taken together, RNA sequencing of the NILs improved the fine-mapping and significantly reduced the number of candidate genes.

### Awns significantly contribute to grain filling

To study the contribution of awns to grain filling under our experimental conditions, we studied the effect of awn removal on seed width, length and weight. Plants of cv. Morex were grown under outdoor conditions and when spikes of these plants emerged the awns were removed. Three groups were made: in the first group, awns were completely removed (no awn), in the second group the awns were cut in half (half awn) and in the third group awns were only cropped at the very tips (full awn) to account for effects by wounding. As an additional control, the thousand grain weight (TGW) and seed width of Morex free from physical injury were also included. Average width of seeds and TGW obtained from Morex spikes with no awn was significantly reduced to, respectively 95.3 and 83.9% when compared to the control (Table 3). These experiments confirm that awn length indeed influences grain filling in barley.

**Table 3:** Average seed width and thousand grain weight measured for plants of cv. Morex or homozygous NILs

Genotype	Awn length (mm)		Seed width (mm)		(g)		Growing season
	Avg^b^ ± SD^c^	%^4^	Avg^b^ ± SD^c^	%^d^	Avg^b^ ± SD^c^	%^d^	
Morex	Control	n.d.	3.6 ± 0.08	100^a^	49 ± 3.2	100^a^	2014
Morex	*Full awn*	[100]	3.6 ± 0.10	101.0^a^	50 ± 2.6	102.3^a^	2014
Morex	*Half awn*	[50]	3.5 ± 0.08	99.3^a^	47 ± 2.2	93.6^ab^	2014
Morex	*No awn*	[0]	3.4 ± 0.10	95.3^b^	41 ± 3.4	83.^c^	2014
BM1 M	151 ± 6	100^a^	3.5 ± 0.05	100^a^	50 ± 1.5	100^a^	2012
BM1 B	130 ± 7	86.1^b^	3.5 ± 0.07	98.0^a^	49 ± 2.1	99.2^a^	2012
DM1 M	156 ± 15	100^a^	3.6 ± 0.04	100^a^	48 ± 0.6	100^a^	2012
DM1 D	107 ± 10	68.6^b^	3.5 ± 0.05	97.2^a^	46 ± 2.0	96.8^a^	2012
BM1-j17 M	189 ± 12	100^a^	3.6 ± 0.12	100^a^	53 ± 5.3	100^a^	2014
BM1-j17 B	161 ± 8	85.9^b^	3.6 ± 0.08	99.4^a^	52 ± 3.3	99.7^a^	2014
DM1-d18 M	188 ± 11	100^a^	3.6 ± 0.05	100^a^	47 ± 1.3	100^a^	2014
DM1-d18 D	134 ± 8	71.3^b^	3.5 ± 0.08	97.2^b^	46 ± 3.5	97.7^a^	2014

^a^Thousand grain weight

^b^Average of biological replicates (*n* ≥ 7)

^c^Standard deviation

^d^Percentage compared to control (=100). Different small letters indicate significant differences between genotypes (one-way ANOVA, *p* ≤ 0.05)

Since this experiment involved a very severe reduction in awn length, we investigated whether the rather moderate changes in awn length observed in our mapping populations also significantly affected grain filling. For this, seeds from homozygous NILs from the mapping populations BM1 and DM1 grown under outdoors conditions in two growing seasons (2012 and 2014) were tested. In both growing seasons, these NILs exhibited significant differences in awn length compared to each other, the B and D allele reduced awn length compared to the M allele to approximately 86 and 70%, respectively. In the DM1 population, a significant reduction in seed width to 97.2% was observed only in the 2014 growing season (Table 3), but no significant differences were found for TGW. The BM1 population exhibited no significant reductions in seed width and TGW.

## Discussion

Although the importance of awn length for grain yield is widely recognised (Bort et al. 1994; Grundbacher 1963; Scharen et al. 1983), little is known about the genes involved in this trait. In this work we used a multiparent mapping population, consisting of six subpopulations to identify awn-length related QTLs. We observed significant variation in awn length, including transgressive segregation in both directions, which argues for the presence of both positive and negative alleles in the parents and/or interactions between loci. Awn length distribution did not differ significantly from normality in the six individual subpopulations, suggesting the involvement of multiple loci. The relatively high broad sense heritability of the trait (*H* ^2^ = 0.75) was in accordance with previous findings that awn length is a highly heritable trait (Taheri et al. 2011).

Most QTL studies in crops have been conducted in bi-parental populations. However, this approach is limited as the small number of parental lines represent only a small portion of the genetic diversity for the trait of interest. Multiparent mapping populations are more genetically diverse than bi-parental populations and allow to screen for effects of multiple alleles per locus. The high genetic diversity combined with increased levels of recombination due to intercrossing increase the resolution and precision of the QTL detection (Cavanagh et al. 2008; Pascuala et al. 2016). To date, only a few multiparent mapping populations using mainly elite cultivars, have been described in wheat and barley (Huang et al. 2012; Mackay et al. 2014; Sannemann et al. 2015). These studies use, respectively, four (Huang et al. 2012) and eight (Mackay et al. 2014; Sannemann et al. 2015) different parental lines as founders for the multiparent mapping population. In our study, we used a multiparent mapping population based on five parental lines; three wild barley genotypes from the eastern and western Fertile Crescent; one barley genotype from the Tibetan highlands in China; and the cultivar Morex. The wild barley parents were selected to maximise genetic diversity in the mapping population. All wild barley lines were crossed with Morex after which one round of backcrossing to the elite parent Morex was used to reduce the proportion of unfavourable wild alleles. As such this intercross population represents a valuable resource for QTL detection, gene identification and as pre-breeding material. Due to the crossing and backcrossing with Morex, on average 75% of the genome of the RILs originate from the recurrent parent Morex. Such asymmetric population structure is not optimal to construct a linkage map as recombination events cannot be observed in large parts of the genome. We, therefore, used the readily available POPSEQ map, which is supported by the genome assembly, as basis for the QTL mapping. We were able to detect 12 significant awn length QTLs from which AL1.1, AL3.1, AL4.1 and AL5.1 co-localise with regions of previously described short awn (*lks*) or *breviaristatum* (*ari*) loci, which are known to exhibit awn length phenotypes (Franckowiak and Lundqvist 2012; Druka et al. 2011). In addition, Morex is a smooth awned barley variety carrying the smooth awn 1 (*raw1*) gene which is located close to AL5.2. To our knowledge, none of the other awn length QTLs including AL7.1, which has the largest effect on awn length, map to previously described loci. The phenotypic variation in both wild and cultivated species suggests that the trait has not been under stringent selection during domestication (Abbo et al. 2014). Three out of four wild barley alleles reduced awn length when compared to Morex, while all accessions carry QTL that have positive as well as negative contribution, explaining the observed transgression.

To refine the mapping of AL7.1 we performed RNA sequencing of two near isogenic lines differing only in the QTL region for HID 382 (DM1-e35). Using this approach, we were able to precisely map the position of the introgression on chromosome arm 7HL. No variants were observed in *HvLks2* or *HvDIM*, where both are located outside of the introgression. In addition, no direct orthologues of known awn-length related genes were identified within the QTL region. Awn length related genes in rice identified so far include: the zinc finger *DROOPING LEAF* (*OsDL*); the auxin response factor *ETTIN2* (*OsETT2*); *awn*-*1* (*an*-*1*) and *an*-*2* which encode, respectively a basic helix-loop-helix protein and the *LONELY GUY LIKE PROTEIN 6* (*OsLOGL6*), which catalyses the final step of cytokinin synthesis; and *LONG AND BARBED AWN1* (*OsLABA1*), which encodes a cytokinin-activating enzyme (Luo et al. 2013; Toriba and Hirano 2014; Gu et al. 2015, Hua et al. 2015). Selection of the candidate genes was done based on the variant calling and expression analysis of the RNA sequencing. At the selected developmental stages (W5.5) a relatively low number of differentially regulated genes were identified in the expression analysis. This can be due to the selected tissue type (entire apex including floral primordia) and/or the relatively early stage of development of the awn. In the variant calling, only MLOC_54434.1, which encodes for a zinc finger (ZF) protein 3 gene, contained a small deletion of 2 nucleotides and co-segregated with the phenotypes of NILs derived from the HIFs DM1 and BM1. Taken together, this MLOC_54434.1 appears to be a promising candidate gene for this major awn length QTL. Noteworthy, two awn length related genes *Lks2* and *DL* also contain a zinc-finger domain (Yuo et al. 2012; Toriba and Hirano 2014). However, phylogenetic analysis indicates that there is no strong homology with MLOC_54434.1 and these previously described awn length related genes.

Previous studies have shown that the photosynthetic activity of the spike in general and the awn in particular are important for grain filling in barley (Abebe et al. 2009; Bort et al. 1994; Scharen et al. 1983; Xue et al. 1997). We have shown that de-awning Morex plants shortly after heading resulted in a significant reduction in seed width and TGW, suggesting that awn length significantly contributes to grain yield. Only a subtle non-significant effect on seed width and TGW was observed DM1 and BM1 NILs. On one hand, this could be due to the rather moderate reduction in awn length in these lines when compared to de-awning of Morex. On the other hand, variation in awn length and correlated differences in net photosynthetic rate of the spike might only become more apparent in stress prone environments with water limitation, high air temperatures, leaf diseases, and other causes of premature leaf senescence (Martin et al. 1993).

Several natural mutant alleles of *lks2* are present in the East Asian barley gene pool (Yuo et al. 2012). Long awns might be seen as hindrance in harvest or storage by the farmer (Yuo et al. 2012). In modern cultivars, the presence of a long awn reduces the value of the barley straw used for feed (Takahashi 1955). However, Morex is known as a smooth-awn malting variety derived from Manchurian barley imported in the early 1920s from Northeast Asia. Morex was characterised by longer awns than three out of the four selected donor barley genotypes. Accordingly, the AL7.1 allele from Morex strongly increased awn length and represents a previously uncharacterised QTL. However, under favourable conditions, only moderate effects on grain size were observed for this QTL.

Taken together, these data exemplify how a multiparent mapping population in barley consisting of wild accessions backcrossed into a commonly used cultivar increased the genetic diversity. Eventually, such approaches may be employed as tool to introduce novel and beneficial traits into the elite barley germplasm.

### **Author contribution statement**

CL, WvE and MK planned the experiments. CL performed experiments and awn length measurements. SE contributed to the development of the mapping population. MM and PH performed SNP data analysis. MB performed QTL analysis. CL, WvE, AW, MvK and MK wrote the manuscript. WvE and AW analysed the RNAseq data. All authors read and approved the final manuscript.

## Electronic supplementary material

Below is the link to the electronic supplementary material.

Supplementary material 1 (XLSX 13 kb)

Supplementary material 2 (XLSX 7208 kb)

Supplementary material 3 (XLSX 11 kb)

Supplementary material 4 (XLSX 1141 kb)

Supplementary material 5 (XLSX 27 kb)

Supplementary material 6 (PDF 4162 kb)
