# Supplementary material for: Fine mapping of a major QTL for awn length in barley using a multiparent mapping population
Source: Theor Appl Genet. 2016 Oct 12;130(2):269–81. doi: 10.1007/s00122-016-2807-y (PMC5263209; doi:10.1007/s00122-016-2807-y)
Supplement: Supplementary file 6 — Supplementary material 6 (PDF 4162 kb) [file 122_2016_2807_MOESM6_ESM.pdf]

Online Resources

Theoretical and Applied Genetics

Fine mapping of a major QTL for awn length in barley using a multiparent mapping population

Corinna B. Liller[1], Agatha Walla[1,2,3], Martin P. Boer[4], Pete Hedley[5], Malcolm Macaulay[5], Sieglinde Effgen[1], Maria von Korff[1,2,3*], G. Wilma van Esse[1,3*], Maarten Koornneef[1,3,6*]

[1]Department Plant Breeding and Genetics, Max Planck Institute for Plant Breeding Research, Carl-von-Linné-Weg 10, 50829 Köln, Germany
[2]Institute for Plant Genetics, Heinrich-Heine-Universität Düsseldorf, Universitätsstr. 1, 40225 Düsseldorf, Germany
[3]Cluster of Excellence in Plant Sciences (CEPLAS), Heinrich-Heine-Universität Düsseldorf, Universitätsstr. 1, 40255 Düsseldorf, Germany
[4]Biometris, Plant Research International, Wageningen University, Droevendaalsesteeg 1, 6708 PB Wageningen, the Netherlands
[5]The James Hutton Institute, Invergowrie, Dundee DD2 5DA, Scotland, UK
[6]Laboratory of Genetics, Wageningen University, Droevendaalsesteeg 1, 6708 PB Wageningen, the Netherlands

[*]Corresponding authors

E-mail: koornneef@mpipz.mpg.de (MK)

Telephone: +49 221 5062 410

Fax: +49 221 5062 413

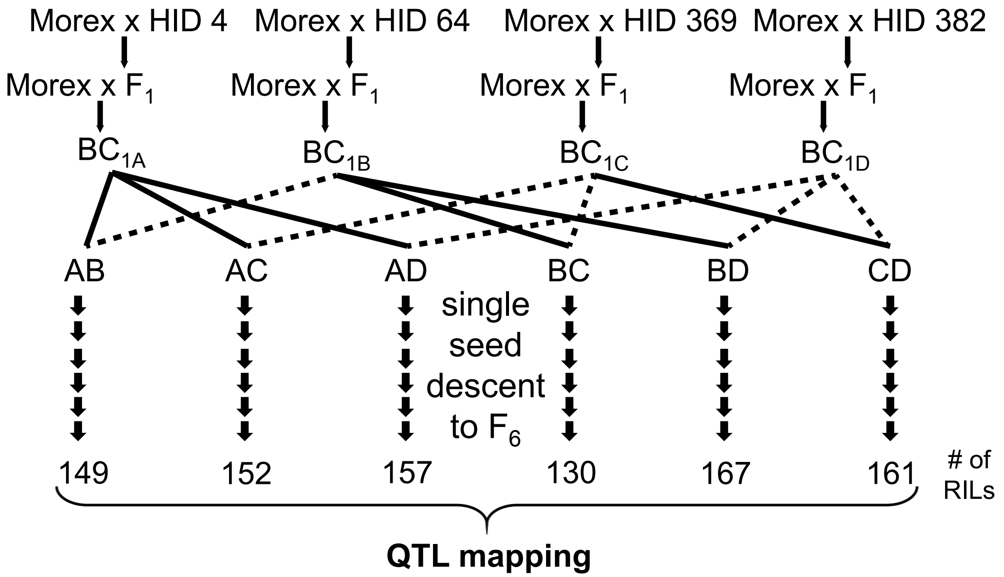

Online resource 1. Crossing scheme of the multiparent recombinant inbred line (RIL) mapping population. To construct the mapping population, each wild barley line was once crossed to the cultivar Morex and the F_1 generation was backcrossed once more to Morex. The resulting backcross generations were then pairwise intercrossed in every possible combination, resulting in six distinct subpopulations, each having three parental lines (Morex and two of the wild barley lines). These subpopulations were propagated by single seed descent until the F_6 generation after the intercross, giving rise to between 130 and 167 recombinant inbred lines (RILs) per subpopulation.

Online Resource 2. HIFs grown under outdoor conditions in 2014

**Family**	**HIF**		**differing at marker(s)**	**generation**
**BM1**	**BM1-v44**	**34-1-3-v44**	2_0117 to 1_0999	F_{10}
	**BM1-e24**	**34-1-3-e24**	2_0117 to 1_0999	F_{10}
	**BM1-j17**	**34-1-3-j17**	i_SCRI_RS_150302/3_1489 to 1_0999	F_{10}
	**BM1-y26**	**34-1-3-y26**	2_0483 to 1_0999	F_{10}
	**BM1-u24**	**34-1-3-u24**	2_0483 to 1_0999	F_{10}
	**BM1-l04**	**34-1-3-l04**	1_0130 to 3_1489	F_9
	**BM1-n01**	**34-1-3-n01**	1_0130 to 2_0483	F_9
**DM1**	**DM1-a41**	**6-2-1-a41**	2_0117 to 1_0999	F_9
	**DM1-s07**	**6-2-1-s07**	2_0117 to 1_0999	F_9
	**DM1-d18**	**6-2-1-d18**	i_SCRI_RS_86194/3_1489 to 1_0999	F_9
	**DM1-e35-R**	**6-2-1-e35-R**	2_0483 to i_SCRI_RS_150302/1_0999	F_{10}
	**DM1-e35**	**6-2-1-e35**	1_1275 to i_SCRI_RS_86194/1_0999	F_{10}

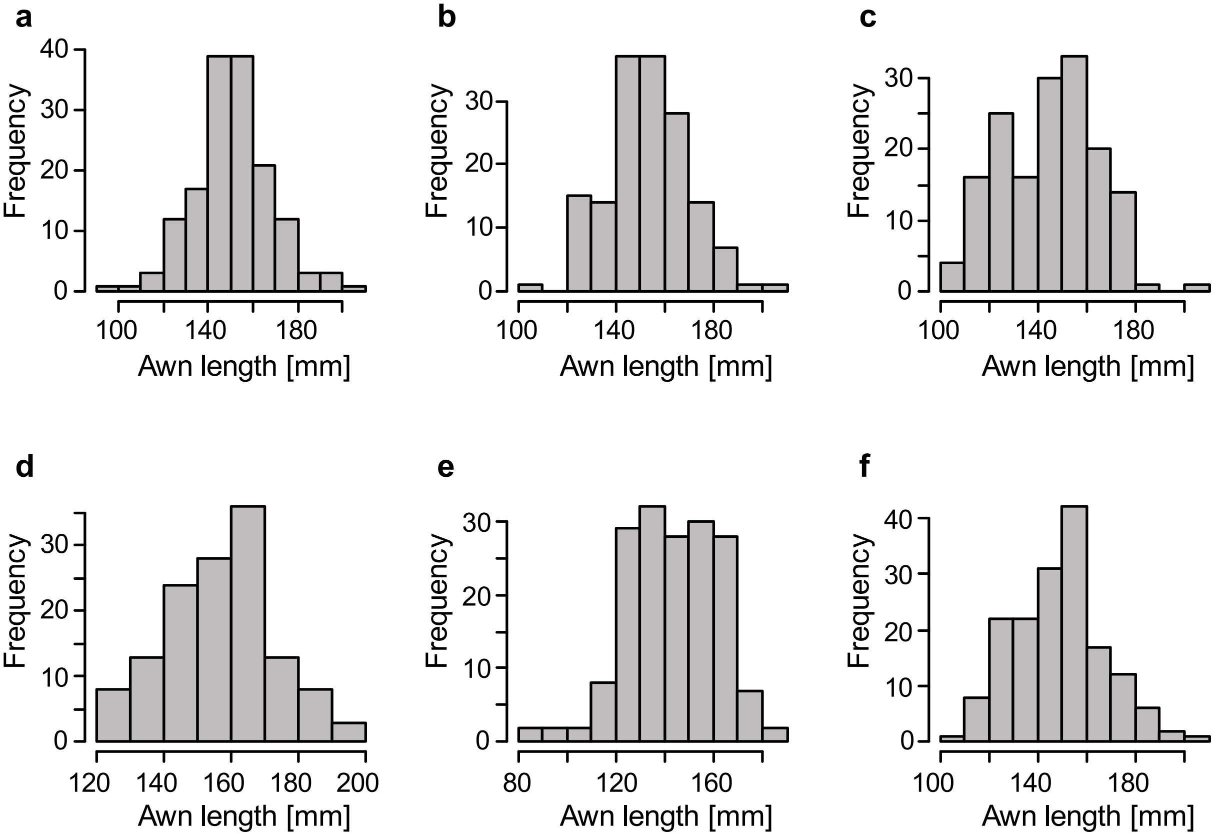

Online resource 4. Distributions of awn length measured in the individual subpopulations. Each individual subpopulation consists of 130 to 165 lines. **a** HID 4/HID 64 [AB], **b** HID 4/HID 369 [AC], **c** HID 4/HID 382 [AD], **d** HID 64/HID 369 [BC], **e** HID 64/HID 382 [BD], and **f** HID 369/HID 382 [CD].

Online Resource 7. Recombination frequency of HIF families used for fine mapping.

family[1]	number of plants					recombination frequency
	total	homozygous Morex	homozygous HID	heterozygous	recombinants	
**BM1**	475	109	111	208	47	5.4
**DM1**	452	117	109	213	13	1.4

[1]Recombination frequencies were based on the recombination between marker the flanking markers 3_0593/1_0999 for BM1 and 2_0483/ 1_0999 for DM1.

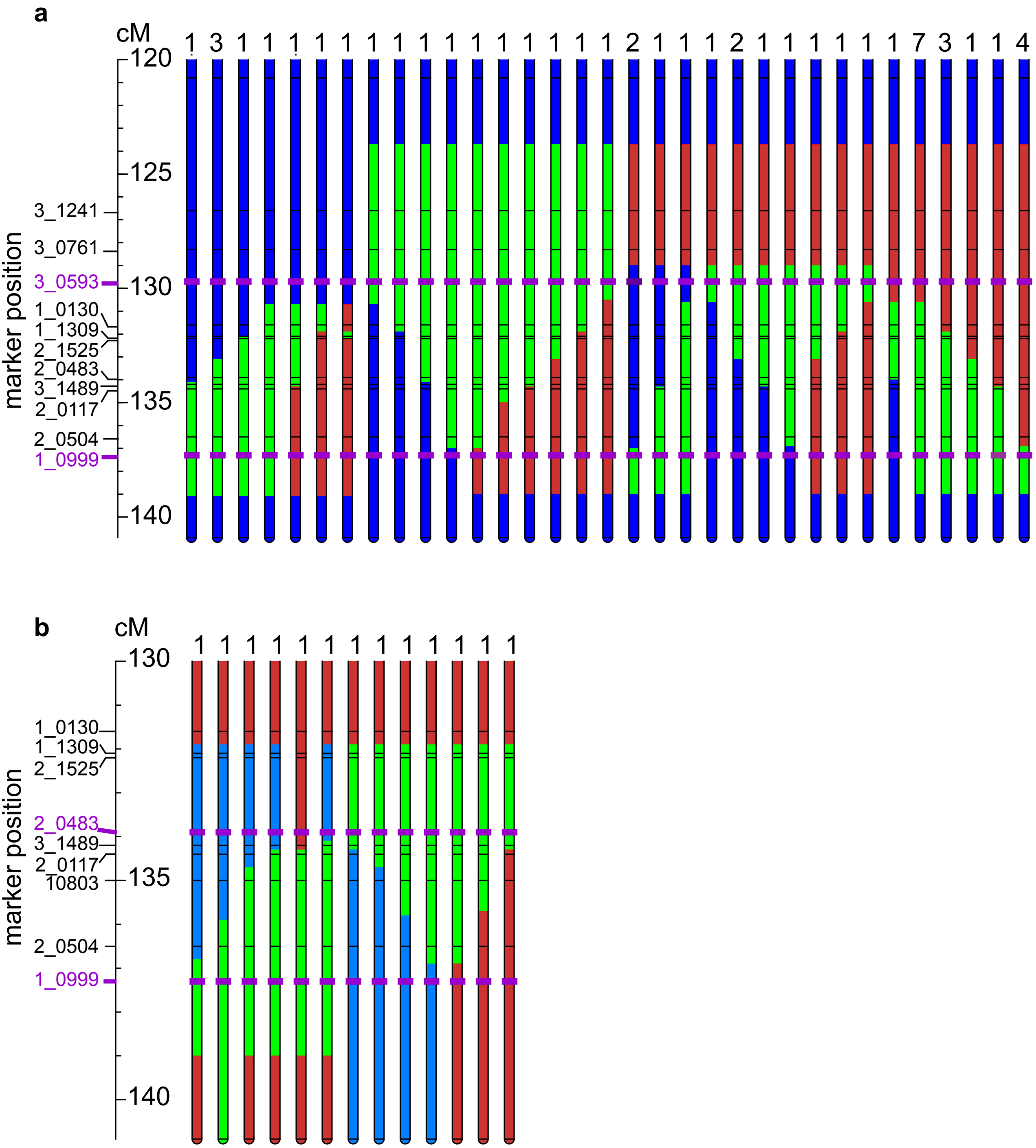

Online resource 8. Graphic representations of the genotypes of recombinants from the **a** BM1 and **b** DM1 fine mapping populations. Map positions are according to the POPSEQ map (Ariyadasa et al., 2014) with modifications to account for actual recombination in these populations and markers missing on the map. Numbers above indicate the number of recombinant individuals with this genotype. Flanking markers are in purple. Colour code: red = Morex, blue = wild barley, green = heterozygous.

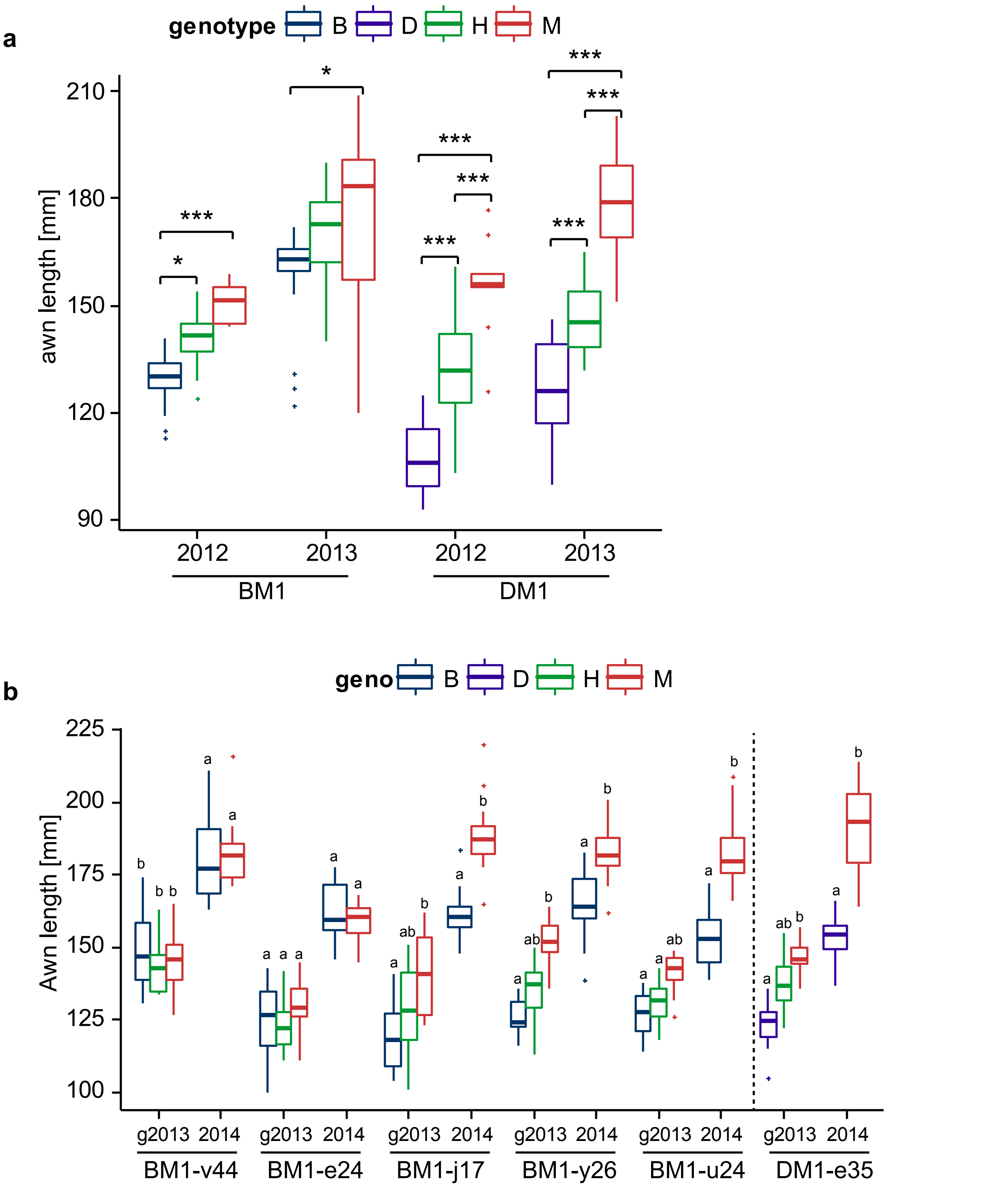

Online resource 9. Awn length in recombinant HIFs grown under outdoor and greenhouse conditions. **a** Boxplots awns length for recombinant HIFs grown under outdoor conditions in 2012 and 2013. Stars indicate significant differences ($p \leq 0.05$ n $\geq$ 30) according to a t-test. **b** Boxplots of awn length measured for recombinant HIFs grown under greenhouse conditions (g2013) and outdoors (2014). Small letters indicate significant differences between genotypes per line (one-way ANOVA; $p \leq 0.05$, n $\geq$ 6).

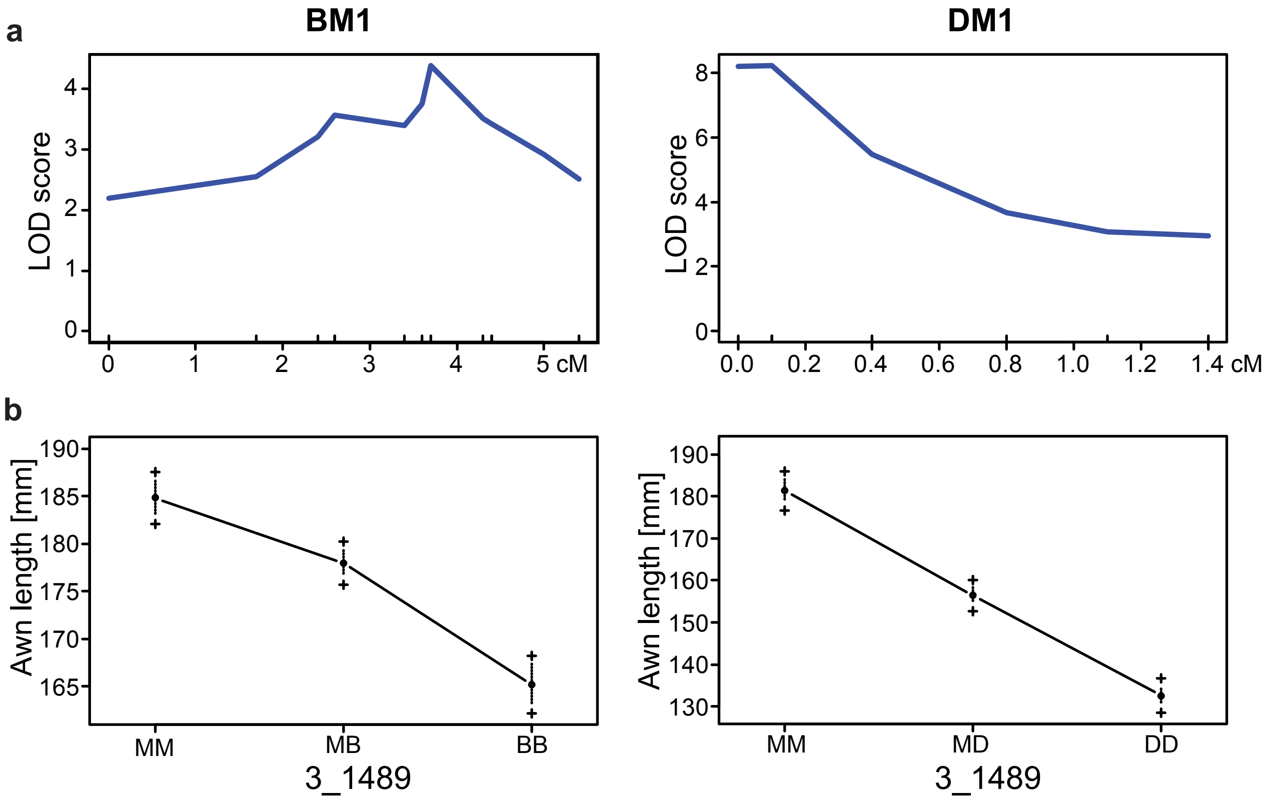

Online Resource 10. Fine mapping and effectplot. **a** QTL mapping results from the fine mapping populations derived from RILs BM1 and DM1. Mapping was performed using the length of the longest awn per plant. Map positions are derived from recombination frequencies of the mapping populations.
Mapping was performed on only the segregating region, treating the populations like F2 generations. **b** Effect plots of the allele effects of homozygous Morex (MM), heterozygous (MB or MD) and homozygous wild barley (BB or DD) on maximum awn length at the position of the most significant marker (3_1489).

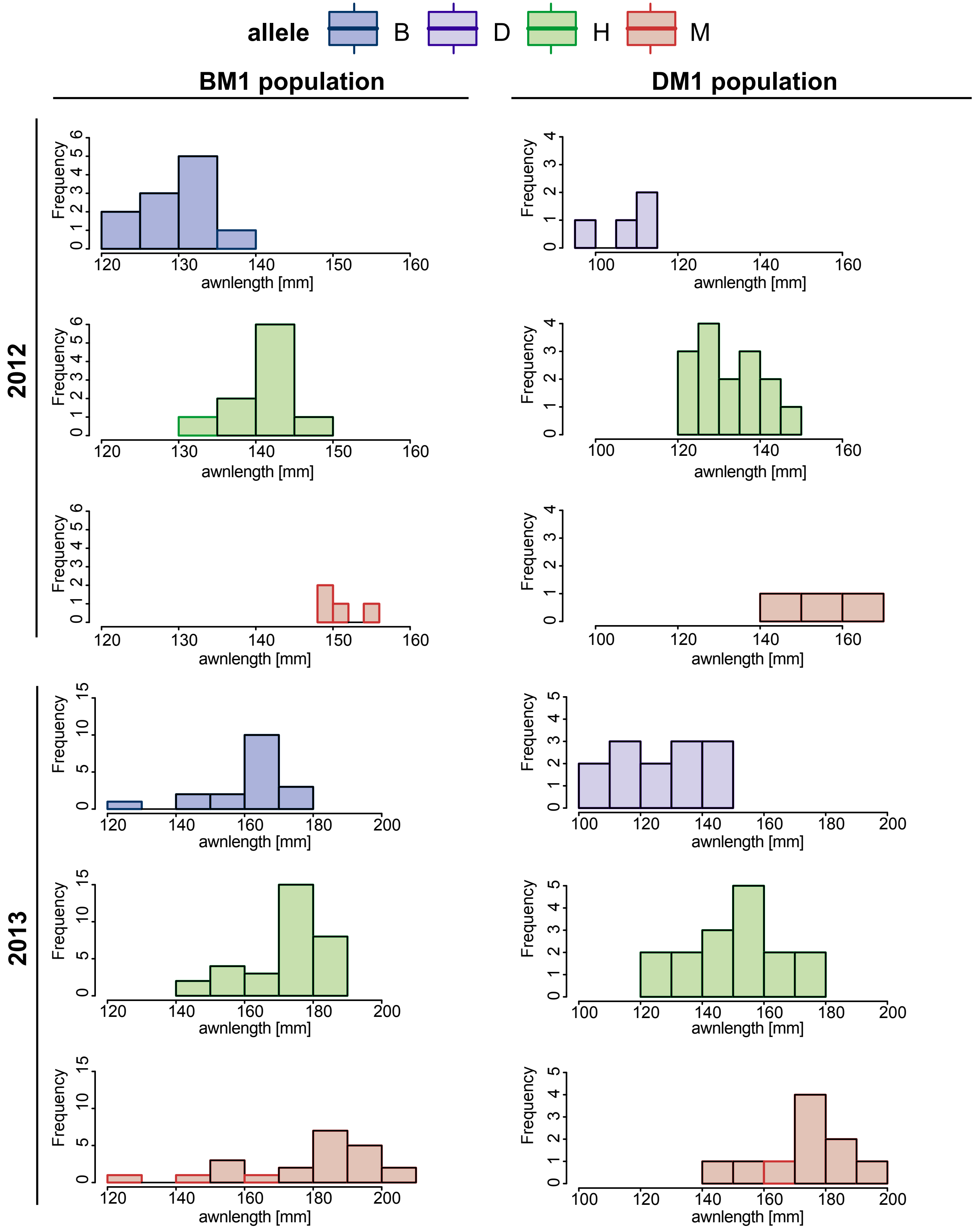

Online Resource 11. Distributions of awn length for the BM1 and DM1 populations. Homozygous and heterozygous alleles are determined based on 3_1489, which did not recombine with the AL7.1 locus.Histograms of the distribution of the trait is shown for different field years/progenies. For 2012 n=25 (BM1)and n=22 (DM1) ; 2013 n= 72 (BM1) and n=39 (DM1).

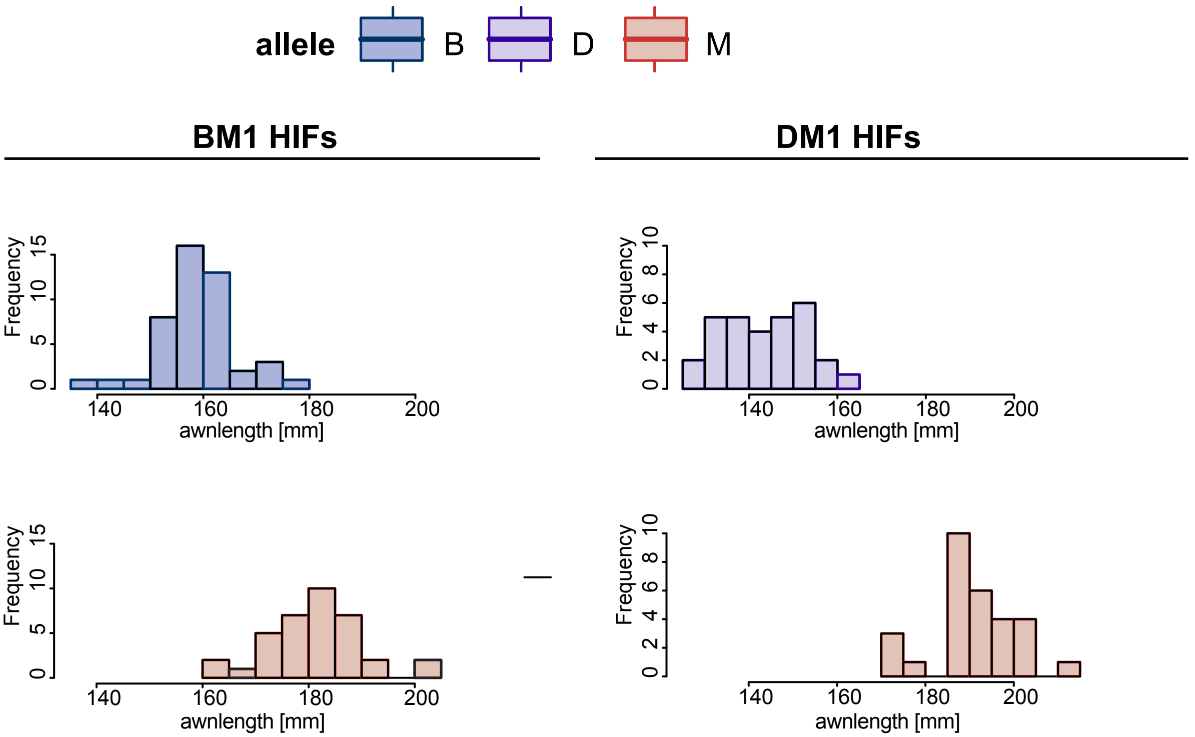

Online Resource 12. Distributions of awn length for the BM1 and DM1 homozygous HIFs used for QTL confirmation. Alleles are determined based on 3_1489, which did not recombine with the AL7.1 locus. n= 82 (BM1) and n=59 (DM1).

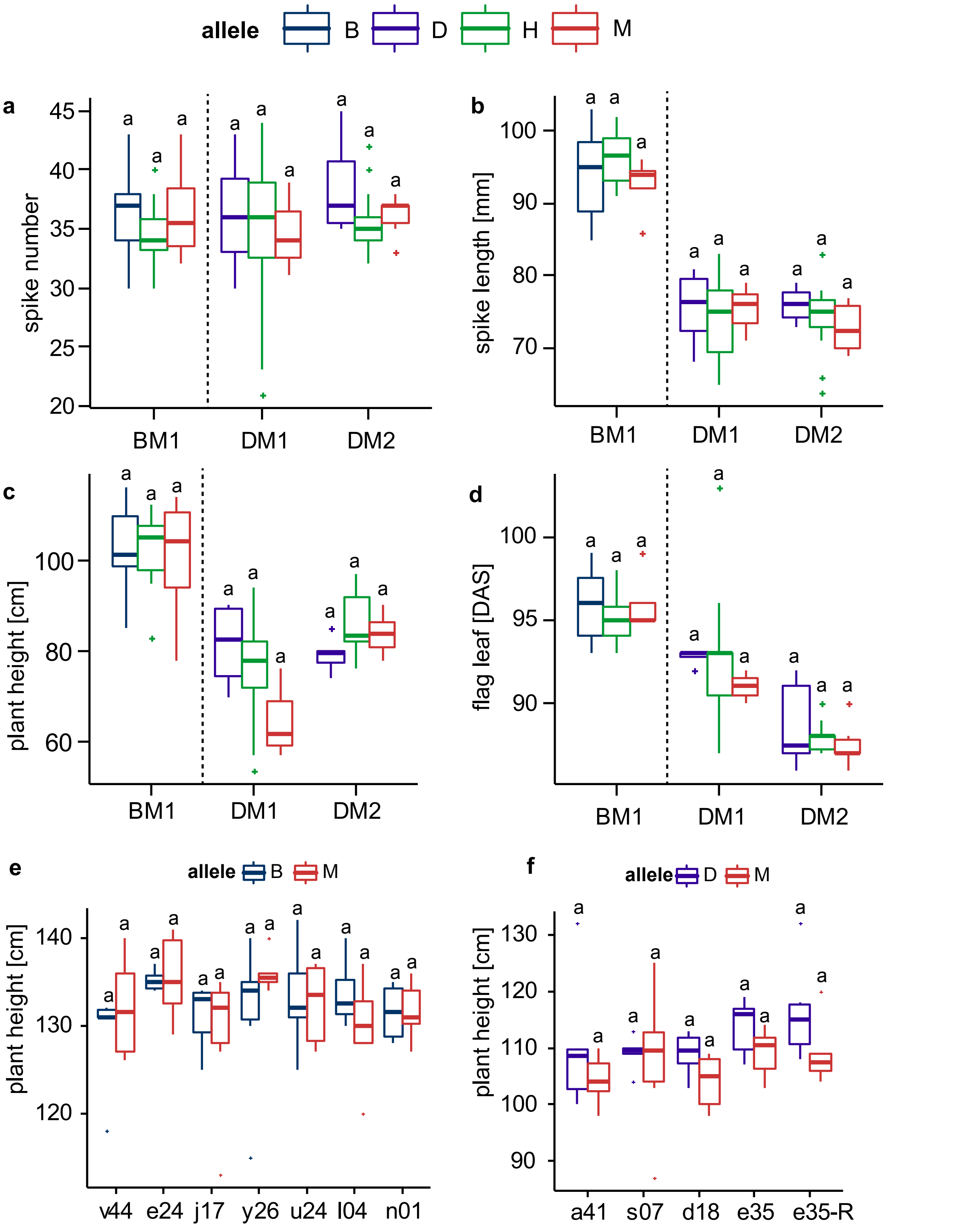

Online resource 13. Trait data of NILs BM1 and DM1. **a-d.** No significant differences in spike length and number, plant height and flowering time were observed (one-way ANOVA, $p \leq 0.05$). Plants were grown under outdoor conditions in 2012. At least 22 plants per NIL were phenotyped and genotyped ($n \geq 4$ plant per allele). **e and f** Heigth measurements were performed under outdoor conditions, 2014. No differences in plant height were observed in either of the NILs (one-way ANOVA, $p \leq 0.05$, $n \geq 5$)

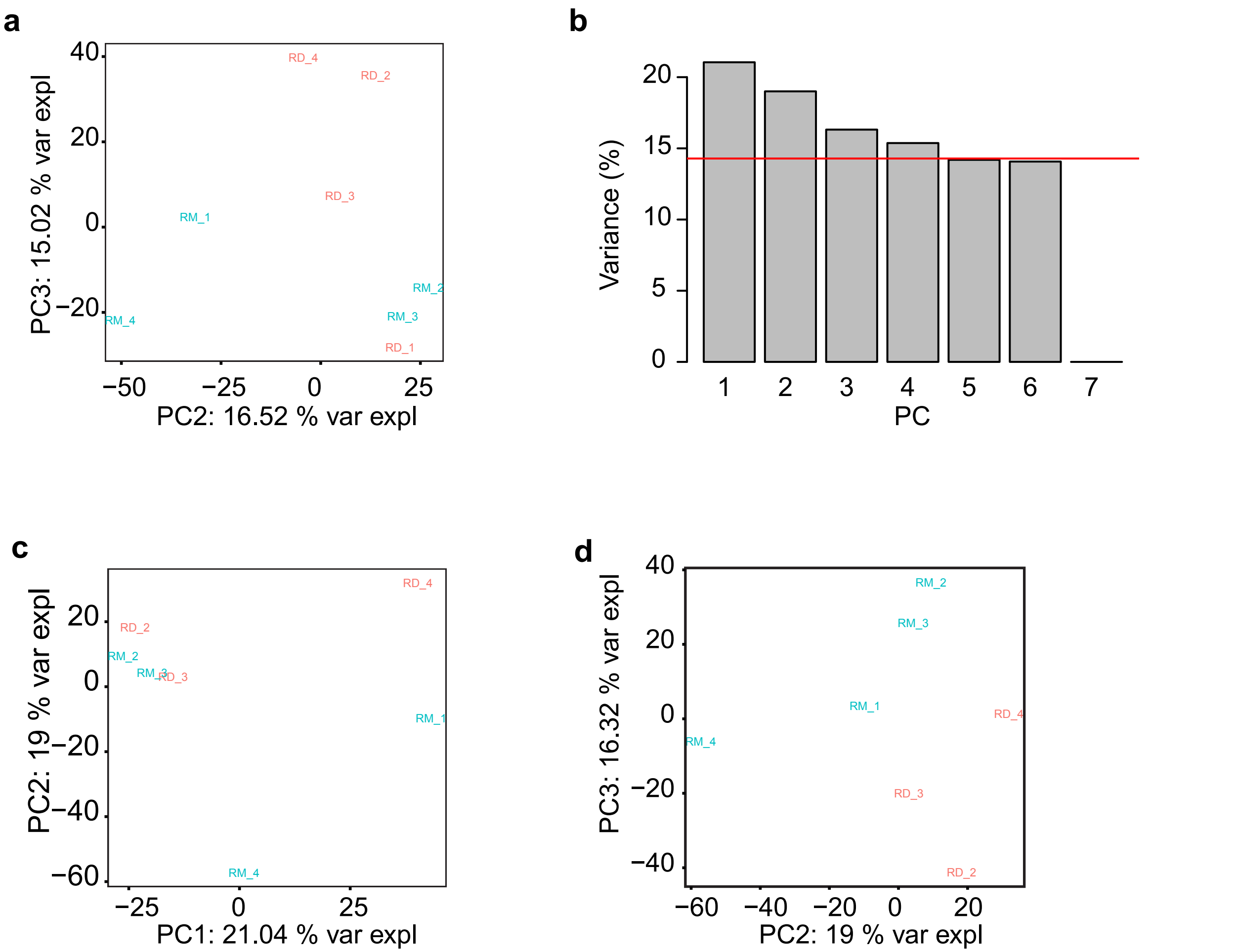

Online resource 16. Variation between different biological replicates used for transcriptional profiling. The variation was based on all expressed transcripts. Blue indicates the HIFs carrying the Morex allele, pink indicates the HIFs that carry the wild allele. Numbers indicate the differend independent biological replicates. **a.** Sample RD_1 was removed as an outlier from the analysis based on a poor correlation with the other RD replicates (visualized in PC2 and PC3). **b.** Scree plot showing which PC explains most of the variability after removal of the outlier RD_1. **c.** PCA for showing PC1 and PC2 which explains respectively 21% and 19% of the variance there is a high variation between the individual biological replicates.
d. PCA for the PC2 and PC3, the latter explains 16 % of the variance. There is a good separation between the different HIFs carrying either the Morex or wild allele.
